# Replantation of Teeth

**Published:** 1873-05

**Authors:** J. O. Smith

**Affiliations:** Babylon, L. I.


### Replantation of Teeth.

An article in the January number of the "Dental Cosmos," on "Replantation of Teeth," leads me to state that in my practice replantation of teeth has nearly ceased to be an experiment. Within the last three years I have successfully performed the operation on five teeth (two for one patient). In each case the tooth was badly decayed, and the root ulcerated. After extracting and treating the tooth socket, I treated the root, and filled not only the cavity but the nerve canal in the root, and replaced the tooth, and without an exception each operation has been a perfect success.

The first patient whose tooth I treated in this way was a young man with whom I was very intimate; he had an ulcer, which gave him much trouble, on the superior incisor. It had been filled several times with different materials without satisfactory results, and he was obliged to have it extracted, and as an experiment I offered to undertake the operation of replacing it, after removing the ulcer and properly filling the tooth. The operation consumed about seventy minutes. There was much sensitiveness about the tooth at first, which soon subsided, and about a year afterward he had the other superior incisor treated in the same manner.

It is now over two years since the last operation, and, to use his own words, "They are the best teeth I have." Since then I have performed the operation on three different patients, and every case has proved a perfect success.—*J. O. Smith, Babylon, L. I., in "Dental Cosmos."*

